# Inverted Takotsubo Syndrome With HELLP Syndrome: A Case Report

**DOI:** 10.3389/fcvm.2022.832098

**Published:** 2022-03-31

**Authors:** Paul Gabarre, Pablo Ruiz, Camille Chenevier-Gobeaux, Etienne Charpentier, Laurie Soulat-Dufour, Ariel Cohen, Laurence Monnier-Cholley, Lotfi Chemali, Hélène François, Mathieu Kerneis, Guillaume Lefèvre, Mathieu Boissan

**Affiliations:** ^1^AP-HP, Hôpital Tenon, Soins Intensifs Néphrologiques et Rein Aigu (SINRA), Paris, France; ^2^AP-HP, Hôpital Tenon, Laboratoire de Biochimie, Paris, France; ^3^AP-HP-Centre Université de Paris, Hôpital Cochin, Department of Automated Biological Diagnostic, Paris, France; ^4^AP-HP, Groupe Hospitalier Pitié Salpêtrière, Unité d'Imagerie Cardiovasculaire et Thoracique ICT, Institut de Cardiologie, Paris, France; ^5^AP-HP, Hôpital Saint-Antoine-Tenon, Service de Cardiologie, Paris, France; ^6^Sorbonne Université, Inserm, Unité de Recherche sur les Maladies Cardiovasculaires, le Métabolisme et la Nutrition, ICAN, Paris, France; ^7^AP-HP, Hôpital Saint-Antoine, Département de Radiologie, Paris, France; ^8^Sorbonne Université, Inserm, UMR_S1155, Paris, France; ^9^Sorbonne Université, ACTION Study Group, INSERM UMRS_1166, Institut de Cardiologie (AP-HP), Paris, France; ^10^Sorbonne Université, Inserm, Centre de Recherche Saint-Antoine, CRSA, Paris, France

**Keywords:** troponin T, troponin I, NT-proBNP, Takotsubo, HELLP syndrome, pregnancy, case report

## Abstract

**Background:**

Takotsubo syndrome is an acute cardiac condition involving sudden, transient apical ballooning of the left ventricle of the heart that may be triggered by emotional stress and some non-cardiac conditions. Its diagnosis is based on clinical presentation, electrocardiogram, cardiac imaging and biomarkers.

**Case Summary:**

Here, we present a novel and original case report of a patient presenting very soon in the post-partum period with an unusual form of Takotsubo syndrome without clinical symptoms of cardiac disease and accompanied by HELLP syndrome. The overall dynamics of the changes in troponin I, troponin T and NT-proBNP levels after delivery were generally similar, but the amount of troponin I was much greater than that of troponin T and troponin I was already elevated before delivery. NT-proBNP levels peaked around the same time as the troponins and the peak concentration was within the same range as that of troponin I.

**Discussion:**

Our findings indicate that assaying circulating cardiac biomarkers, especially troponin I and NT-proBNP, may be a useful complement to non-invasive cardiac imaging including transthoracic echocardiography and cardiovascular magnetic resonance imaging, in the diagnosis of Takotsubo syndrome. They illustrate the importance of cardiac biomarkers in assisting diagnosis of this disease.

## Introduction

Takotsubo syndrome is a sudden, transient, and acute dysfunction of the left ventricle of the heart that can also involve the right ventricle. It is frequently preceded by physical or emotional stress. Takotsubo syndrome affects women more often than men and, commonly, 60–75-year-olds; thus, at onset, it can be mistaken for acute coronary syndrome. Although its exact pathophysiology is unknown, the main hypothesis is that it is due to exaggerated sympathetic stimulation, inducing a catecholamine excess, which seems to be more increased in Takotsubo syndrome than in acute myocardial infarction (1).

The effect of Takotsubo syndrome on circulating cardiac biomarkers is controversial. Two types of cardiac biomarker—natriuretic peptides and cardiac troponins—are used in the diagnosis of both Takotsubo syndrome and acute myocardial infarction (1, 2). In the International Takotsubo Diagnostic Criteria (InterTAK Diagnostic Criteria), levels of cardiac biomarkers, including troponin, are described as moderately elevated in most cases whereas levels of brain natriuretic peptide (BNP) are substantially elevated. Some exceptions have been reported, however (1, 3).

Here, we describe the clinical and biological features as well as non-invasive cardiac imaging of an unusual case of peri-partum HELLP (hemolysis, elevated liver enzymes, low platelet) syndrome complicated by an inverted Takotsubo syndrome. This patient was asymptomatic for cardiac disease, the twelve-derivation electrocardiogram demonstrated no pathological features, and the amount of troponin I was strongly increased to within the same concentration range as the N-terminal prohormone of BNP (NT-proBNP). Our interpretation of the biological test results illustrates the potential use of these cardiac biomarkers in assisting diagnosis of Takotsubo syndrome.

All patient-specific information was anonymized.

## Methods

Plasma levels of cardiac high-sensitivity troponin I (cTnIhs) and troponin T (cTnThs) in samples collected in lithium heparin were assayed by using the Architect *ci* 8200 (Abbott) and Cobas E801 (Roche Diagnostics) analyzers, respectively. Normal values of cTnIhs and cTnThs (women cTnIhs 99th percentile, cTnThs overall population) were <15.6 and <14 ng/L, respectively (manufacturer's data). Plasma levels of N-terminal prohormone of brain natriuretic peptide (NT-proBNP) were assayed by using the Cobas analyzer (women 97.5th percentile value <254 ng/L). Calculation of molar concentrations of cardiac biomarkers was based on the following estimated molecular masses: troponin T, 37 kDa; troponin I, 24 kDa; NT-proBNP, 8.5 kDa.

## Case Presentation

A 38-year-old pregnant woman (gravida 2, para 0) was admitted at 40 weeks of gestation to our labor and delivery unit with regular contractions after an uncomplicated, regularly supervised pregnancy. She reported a history of mild asthma, endometriosis, and an early miscarriage. At admission, physical examination showed normal vital signs and a fully dilated cervix. Laboratory results were unremarkable. She had an uncomplicated spontaneous vaginal delivery with Apgar scores of 10 and 10. A blood pressure of 152/67 mmHg was noted once during labor.

The patient received extremely stressful news about her child just after delivery. Thirty hours after delivery, she reported a sudden and intense headache associated with epigastric pain. She had no chest pain or respiratory discomfort, such as dyspnea, and neurological examination found no abnormalities. Laboratory tests revealed cytolysis (ASAT, 414 U/L and ALAT, 399 U/L; LDH, 1,017 U/L), associated with a slightly low haptoglobin concentration (0.44 g/L). A whole blood count showed thrombocytopenia (84 G/L) but no evidence of disseminated intravascular coagulation. Slight signs of inflammation were present (white blood cells count, 12.9 G/L; CRP, 77 mg/L). The blood creatinine concentration was elevated (1.12 mg/dl or 85 μmol/L) and was associated with *de novo* proteinuria. The urinary protein profile demonstrated tubular and glomerular involvement (total proteinuria, 0.45 g/mmol creatininuria; albumin, 227 mg/mmol creatininuria; alpha-1-microglobulin, 9.03 mg/mmol creatininuria; alpha-2-macroglobulin, 2.27 mg/mmol creatininuria; IgG, 60.43 mg/mmol creatininuria; retinol-binding protein, 0.53 mg/mmol creatininuria; transferrin, 37.90 mg/mmol creatininuria). We diagnosed HELLP (hemolysis, elevated liver enzymes, low platelet) syndrome, a complication of pregnancy with a risk of kidney failure. The patient was admitted to the nephrology intensive care unit on day 2.

**Figure 1:**
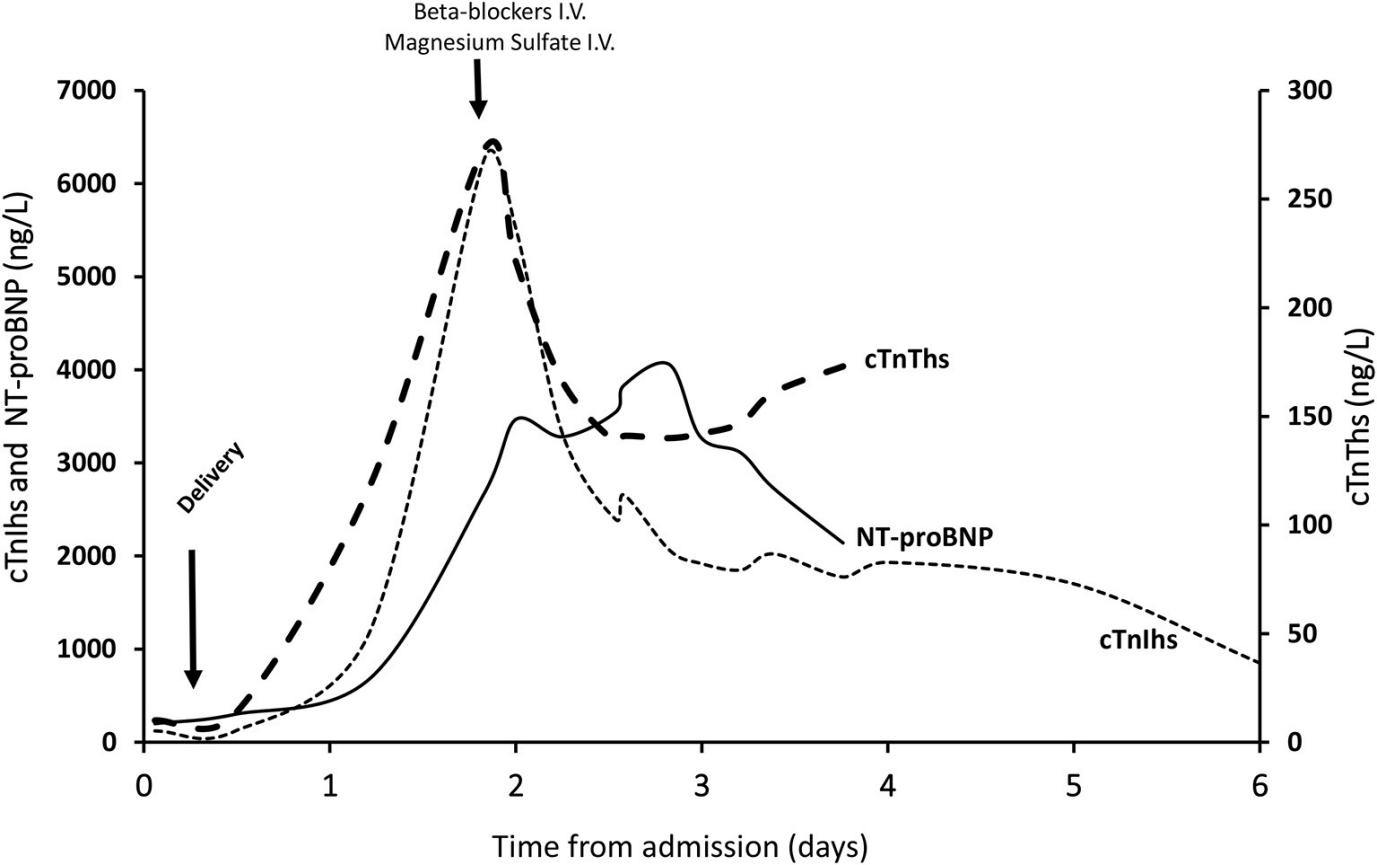
Changes in the cardiac biomarkers cTnIhs, cTnThs, and NT-proBNP. Concentrations of circulating cardiac biomarkers were measured upon admission to hospital (day 0) and at intervals until to discharge (day 6). They are presented as ng/L. Delivery occurred between day 0 and 1 after admission. Intravenous (i.v.) magnesium sulfate and the beta-blocker labetalol were administered as indicated.

On admission to the nephrology intensive care unit, we found elevated levels of cTnIhs and cTnThs, indicating cardiac necrosis, as well as elevated levels of NT-proBNP. Retrospective assays of cTnIhs and cTnThs in stored plasma samples revealed that pathological concentrations of cTnIhs (i.e., above the 99th percentile threshold) were already present 1 h before delivery but not of cTnThs (Figure 1). Measurements of these cardiac biomarkers up to 6 days after delivery found distinct qualitative and quantitative differences. Qualitatively, whereas pathological levels of cTnIhs were already present before delivery, cTnThs began to increase 0.5 day later. Quantitatively, between admission and day 6, cTnIhs concentrations ranged from 115 to 6,264 ng/L whereas cTnThs concentrations were much lower, ranging from 10 to 273 ng/L; NT-proBNP concentrations ranged from 200 to 4,056 ng/L. Both cTnIhs and cTnThs reached a peak concentration at day 2 and decreased soon after. The peak concentration of cTnIhs, however, was much greater than that of cTnThs (median cTnIhs/cTnThs molar ratio = 20.9; 95% C.I., 14.9–36.6—see Methods Section). NT-proBNP levels peaked around the same time as those of the troponins but decreased only after day 3.

**Figure 2:**
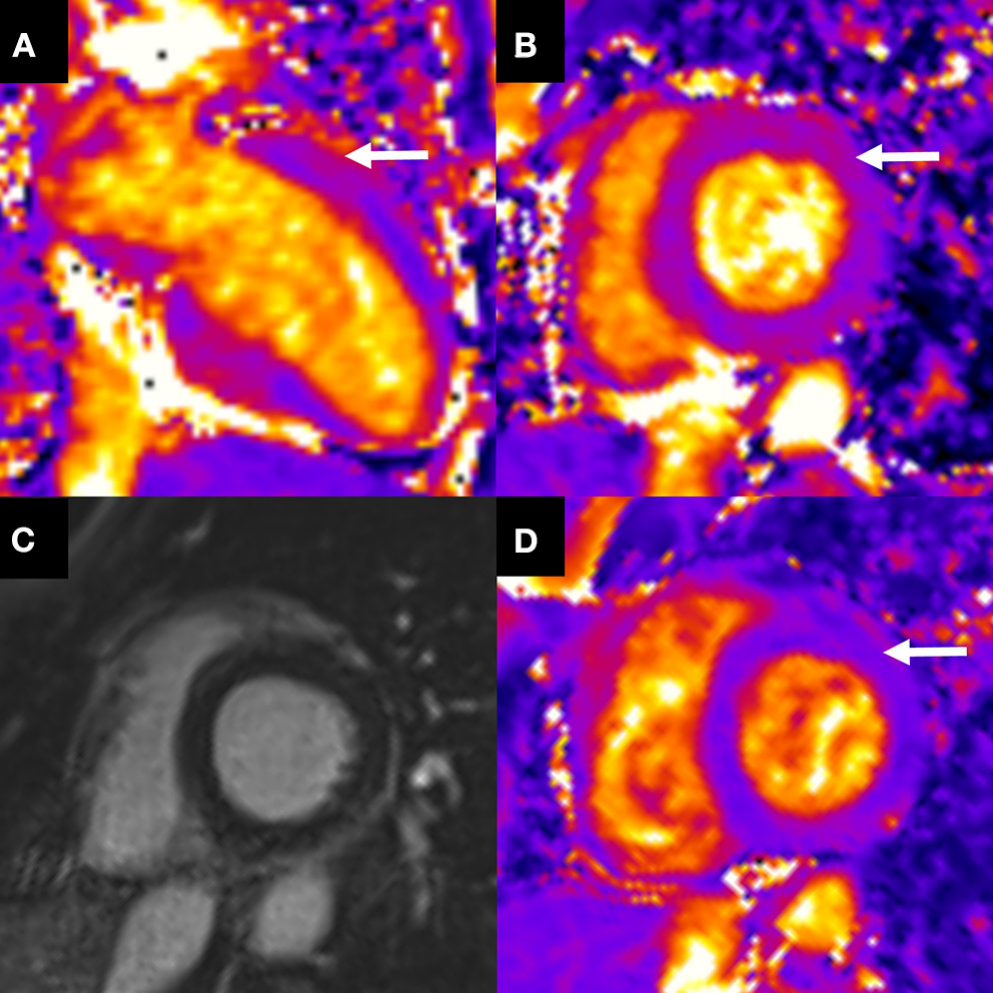
Initial cardiac MRI and follow-up. Cardiac MRI at day 3 after transfer to the nephrology intensive care unit, showed **(A,B)** an elevation of basal T2 mapping value (60 ± 5 ms) related to myocardial edema (arrows) not involving the mid and apical left ventricle without late gadolinium enhancement **(C)**. **(D)** One month later, short axis T2 mapping showed regression of the myocardial edema with a normal T2 mapping value (49 ± 1 ms) (arrow).

The twelve-derivation electrocardiogram was unremarkable. No coronary stenosis or dissection was found by coronary computed tomographic angiography. Transthoracic echocardiography performed at day 1 after transfer to the nephrology intensive care unit (i.e., at day 3 post-delivery) revealed hyperechogenicity, ballooning and akinesia of the basal and mid segments of the inferoseptal (Supplementary Video 1), inferior (Supplementary Video 2) and anteroseptal (Supplementary Video 3) walls, with mild impairment of left ventricular ejection function (LVEF, 45%), suggesting inverted Takotsubo syndrome, a rare variant of this disease that presents with basal ballooning instead of apical ballooning. Cardiac MRI performed at day 3 after transfer to the nephrology intensive care unit (i.e., at day 5 post-delivery) revealed a non-dilated, non-hypertrophied left ventricle, slightly altered global LVEF (51%) and characteristic wall motion abnormalities of inverted Takostubo syndrome including hypokinesia of the basal third predominating over the septum and apical hyperkinesia (Supplementary Video 4) associated with diffuse basal myocardial edema detected by T2 mapping sequence without late gadolinium enhancement (Figures 2A–C). Global T2 mapping values demonstrated a gradient from the base to the apex of the left ventricle: 56 ± 4 ms in the first third, 51 ± 7 ms in the second third, and 48 ± 3 ms at the apical level.

The patient was treated with an intravenous beta-blocker (labetalol) as soon as the diagnosis of HELLP syndrome was made and with intravenous magnesium sulfate until abolition of neurological signs. Her evolution was favorable, with a reversal of headache, hepatic cytolysis, acute renal failure and thrombocytopenia. In addition to the beta-blocker, an angiotensin-converting enzyme inhibitor (captopril) was used at day 3 to improve blood pressure control with no adverse events. cTnIhs levels slowly decreased to 849 ng/L on day 6 when she was discharged from the nephrology intensive care unit. One month after discharge, the patient was in complete remission of her post-partum inverted Takotsubo syndrome. A follow-up cardiac MRI demonstrated complete resolution of left ventricular dysfunction (LVEF, 60%) and regional left ventricular function recovery (Supplementary Video 5). Moreover, normalization of T2 mapping values indicated the disappearance of myocardial edema (in Figure 2D): 50 ± 5 ms in the first third, 46 ± 6 ms in the second third, and 46 ± 5 ms at the apical level. These findings from cardiac MRI were consistent with our diagnosis of inverted Takotsubo syndrome. Three months after discharge, medical examination found a normal blood pressure of 123/82 mmHg and blood analyses were normal. The main results of the case presentation were summarized in Supplementary Figure 1.

## Discussion

Here, we describe a case of Takotsubo syndrome in a 38-year-old woman, which occurred post-partum after spontaneous vaginal delivery. This case is novel and interesting, first, because it occurred very soon after childbirth, second, because of the unusual clinical features and, third, because it was accompanied by HELLP syndrome. Our measurements of elevated levels of circulating cardiac biomarkers illustrate the importance of these biomarkers in assisting diagnosis and treatment of this condition.

Although the definitive diagnosis of Takotsubo syndrome was made only several days post-partum, retrospective assays of circulating troponins revealed myocardial damage occurring at the same time as or prior to childbirth. Reports of changes in blood cardiac biomarker levels during pregnancy, delivery and post-partum in the literature are rare. According to one study, changes in cardiac morphology occur within 1 week post-partum, but not during pregnancy (4). Using the same assay for cTnIhs as that used in our study, that study found only slightly but statistically significant elevated levels of cTnIhs at the end of pregnancy (2 ng/L, i.e., 0.07-fold > normal threshold) when compared those observed during the first trimester. In our case, by contrast, the first cTnIhs measurement 1 h before delivery was already substantially increased (120 ng/L, i.e., 7.7-fold > normal threshold) probably due to early cardiac injury.

The case of our patient has unusual clinical features: she was asymptomatic for cardiac disease, the twelve-derivation electrocardiogram demonstrated no pathological features, and the amount of cTnIhs was strongly increased to within the same concentration range as NT-proBNP. The findings from ultrasound and MRI imaging were characteristic of a rare form of the disease: inverted Takotsubo syndrome, in which there was severe basal hypokinesia and a moderate apical hyperkinesia. Takotsubo syndrome is a complex, acute cardiac condition that resembles acute coronary syndrome, usually occurring in the absence of obstructive coronary artery disease and leading to regional left ventricular wall motion abnormality and an impairment of left ventricular contractility (5). The area most often affected is the apical region of the left ventricle (6); in some cases, however, as in our case, the basal area of the left ventricle is affected (7).

In addition to Takotsubo syndrome, the patient presented with HELLP syndrome. The simultaneous occurrence of Takotsubo syndrome in patients with HELLP syndrome has been reported in only a few other cases (8). In a study of 10 cases of post-partum Takotsubo syndrome, two women (20%) presented with HELLP syndrome and five (50%) with preeclampsia (9).

Our understanding of the literature is that the precise pathophysiology of Takotsubo syndrome is unknown; several different pathophysiological mechanisms probably act, individually or synergistically, to cause Takotsubo syndrome. Moreover, it appears that it is often difficult to assess whether any observed modification of a signaling pathway is a cause or a consequence of the episode of Takotsubo syndrome. Sympathetic hyperstimulation is a crucial pathophysiological feature, and catecholamines and G_i_α signaling pathways at the level of β2 adrenergic receptor have an important role, resulting in acute myocardial inflammation, myocardial lipotoxicity, low energy production, and NO synthesis, which are hallmarks of Takotsubo syndrome (10, 11). Other possible mechanisms include disruption of calcium regulation, generalized endothelial dysfunction, and oxidative stress (10, 11).

Although the exact pathophysiology of HELLP syndrome has not been clearly defined, we have identified several similarities between the documented mechanisms of HELLP and Takotsubo syndromes, including systemic endothelial dysfunction, strong involvement of oxidative stress, and a systemic inflammatory response (12). A possible link between these two diseases should be evaluated by future studies. One plausible hypothesis to explain the possible link between HELLP and Takotsubo syndrome is an interplay between endothelial abnormalities as recently discussed (13). In HELLP syndrome and during pre-eclampsia there is an imbalance of vasoconstriction and vasodilation mediators mainly at the placental level, and in Takotsubo syndrome dysfunctions in the microcirculation have been documented (14), suggesting that abnormal regulation of vasoconstriction/vasodilation in the microcirculation may underlie both syndromes.

The diagnosis of Takotsubo syndrome is based partly on clinical data, but some biological signs, such as cardiac biomarker changes, have been identified as diagnostic tools (15). No definitive specific or sensitive biomarker for the disease has been proposed, although several biomarkers such as copeptin, lipid profile, sLOX-1, ischemia-modified albumin, sST-2, and chromogranin-A have been proposed to distinguish it from acute coronary syndrome (16, 17). Little is known about cardiac biomarker changes during the acute and remission phases of Takotsubo syndrome (18).

The main cardiac biomarkers reported to be modified in Takotsubo syndrome are NT-proBNP and BNP (19). There is a significant and persistent elevation of NT-proBNP and BNP levels during the acute phase of the disease, which correlates with both the elevation of catecholamines and the severity of left ventricular systolic dysfunction (20). BNP levels ≥238 ng/L and the absence of calcium channel blocker use are independent risk factors for delayed recovery, whereas a leptosomic build (BMI <20 kg/m^2^) is an independent predictor of rapid recovery (21). This increase in BNP levels is more substantial than that of cTnThs in Takotsubo syndrome patients (22). In our case, the increase of circulating troponin isoforms was unusual. The increase in cTnIhs was greater than the increase in cTnThs, as already reported (23), and was within the same range as NT-proBNP; however, whereas pathological levels of cTnIhs were already present before delivery, cTnThs began to increase later.

Some authors have reported similar changes in troponins during acute coronary syndrome and Takotsubo syndrome, which may result in a misdiagnosis (22). Acute coronary syndrome and Takotsubo syndrome overlap significantly in their clinical presentations and Takotsubo syndrome is often mistaken for acute anterior wall ST-segment elevation myocardial infarction (due to an occlusion of the proximal left anterior descending artery). In our case, the ratio of NT-proBNP (ng/L) to cTnThs (μg/L) at peak was 9,798. This was greater than the threshold level of 5,000 indicating Takotsubo syndrome rather than acute anterior wall ST-segment elevation myocardial infarction (24).

Whereas, the dynamics of the changes in cTnIhs, cTnThs and NT-proBNP in the days following delivery were similar, the amount of cTnIhs was much greater than that of cTnThs (at peak, 400-fold > normal threshold for cTnIhs compared to 25-fold > normal threshold for cTnThs). One caveat to these measurements is that post-translational modifications of troponins upon cardiac necrosis may affect the quantification of circulating forms of the proteins (25); thus, our observations should be substantiated by quantification of all circulating forms of troponins throughout the duration of the disease. Although no definitive conclusion can be drawn at the moment about the comparative sensitivity of cTnIhs or cTnThs for optimal cardiac monitoring during Takotsubo syndrome, our findings suggest that monitoring cTnIhs and/or NT-proBNP would be more useful than monitoring cTnThs because cTnIhs levels increase sooner and much more than cTnThs levels.

In the case presented here, the onset of Takotsubo syndrome post-partum occurred soon after spontaneous vaginal delivery, whereas it generally occurs several days later (26). Takotsubo syndrome is much more common after cesarean section than after vaginal delivery because several risk factors are associated with it, including the psychological and physical stress associated with cesarean section, acute pain and bleeding causing increased catecholamine levels, and use of uterotonic or tocolytic treatments (26, 27). Takotsubo syndrome is often associated with a preceding stressful physical or emotional event (5); physical triggering factors are more prevalent than emotional triggers. The absence of triggering factors does not preclude the diagnosis of Takotsubo syndrome, however. Interestingly, our patient received extremely stressful news about her child just after delivery, which may have contributed to triggering Takotsubo syndrome. The patient received no exogenous drugs, notably, no catecholamines or sympathomimetic drugs that might have precipitated the episode of Takotsubo syndrome.

Because the pathophysiology of Takotsubo syndrome is not precisely understood, there are no well-established guidelines for treating and managing this condition. We treated our patient with angiotensin-converting enzyme (ACE) inhibitors to address the observed abnormal left ventricle wall motion and impaired LVEF, and with a beta-blocker to prevent the potential effects of an adrenergic surge. The use of angiotensin-converting enzyme inhibitors to treat Takotsubo syndrome is associated with improved survival and fewer recurrent events (5, 28). The effectiveness of beta-blockers, by contrast, is less clear. Whereas, observational studies and meta-analyses have found that short- and long-term treatment with beta-blockers is not beneficial in reducing mortality or preventing recurrence (5, 28–31), a recent state-of-the-art review (32) recommended treating patients with Takotsubo syndrome with both angiotensin-converting enzyme inhibitors and beta-blockers. Specifically, they recommended carvedilol, which is a non-cardioselective beta-blocker. In our case, we used labetalol, which is also a non-cardioselective beta-blocker, because our patient wished to breastfeed her baby. Beta-blockers in breast milk can cause hypotension, bradycardia, and hypoglycemia in the infant (33). According to the *Centre de Référence sur les Agents Tératogènes* in France, there are no data concerning the secretion of carvedilol into breast milk, but secretion of labetalol into breast milk is weak and it is estimated that the infant receives <1% of the maternal dose. Moreover, the half-life for elimination of labetalol is short (4 h), whereas that of carvedilol is substantially longer (7–10 h).

Takotsubo syndrome was originally thought not to be a life-threatening disease (34), however, more recent studies have found higher mortality rates in Takotsubo syndrome patients than expected due to long-term mortality, which surpasses that of patients with ST-segment elevation myocardial infarction (35). Moreover, there are reports that the initial presentation can be associated with fatal complications, including cardiogenic shock, congestive heart failure, and lethal arrhythmias, leading to an in-hospital mortality of 2.0–8.7% (5, 36). Thus, the prognosis of Takotsubo syndrome ranges from rapid recovery to poor early and long-term outcomes. A multicenter study of over 1,000 patients from the German and Italian Stress Cardiomyopathy (GEIST) registry yielded four variables as independent predictors of in-hospital complications: a history of neurologic disorders, right ventricular involvement, reduced LVEF, and male sex (36). Therefore, a GEIST prognostic score may be helpful in early risk stratification. Another analysis of the GEIST registry revealed dyspnea at admission as an independent risk factor for in-hospital complications and poor long-term outcomes (37). Also, in-hospital complication rates and long-term mortality were reported to be similar in typical and atypical types of Takotsubo syndrome (38). Our patient recovered full cardiac function 1 month after delivery with no cardiac complications. Consistent with this rapid recovery, she presented good prognostic factors: no neurologic disorder, no right ventricular involvement, only mild impairment of LVEF, and was a female presenting with no dyspnea at the onset of the episode of Takotsubo syndrome.

## Conclusion

We describe an unusual case of Takotsubo syndrome in a post-partum woman after spontaneous vaginal delivery. This patient was asymptomatic for cardiac disease, the twelve-derivation electrocardiogram demonstrated no pathological features, and the Takotsubo syndrome was accompanied by HELLP syndrome. The overall dynamics of the changes in troponin I, troponin T and NT-proBNP levels after delivery were similar, but the amount of troponin I was much greater than that of troponin T and troponin I was already elevated before delivery. The magnitude of the increase of troponin I was similar to that of the increase of NT-proBNP. Our findings indicate that assaying circulating cardiac biomarkers, especially troponin I and NT-proBNP, may be a useful complement to the diagnosis of Takotsubo syndrome by non-invasive cardiac imaging. They illustrate the importance of cardiac biomarkers in assisting diagnosis of this disease.

## Data Availability Statement

The original contributions presented in the study are included in the article/Supplementary Material, further inquiries can be directed to the corresponding author/s.

## Ethics Statement

Written informed consent was obtained from the individual for the publication of any potentially identifiable images or data included in this article.

## Author Contributions

PG and HF performed physical examination. PR, CC-G, and LC performed biological analysis. EC, LS-D, AC, LM-C, and MK performed imaging. PG, PR, GL, and MB wrote the manuscript. All authors contributed to the article and approved the submitted version.

## Conflict of Interest

The authors declare that the research was conducted in the absence of any commercial or financial relationships that could be construed as a potential conflict of interest.

## Publisher's Note

All claims expressed in this article are solely those of the authors and do not necessarily represent those of their affiliated organizations, or those of the publisher, the editors and the reviewers. Any product that may be evaluated in this article, or claim that may be made by its manufacturer, is not guaranteed or endorsed by the publisher.

## Supplementary Material

Supplementary Video 1 Transthoracic echocardiography, apical four-chamber view. Hyperechogenicity, ballooning and akinesia of the basal and mid segments of the inferoseptal wall.

Supplementary Video 2 Transthoracic echocardiography, apical two-chamber view. Hyperechogenicity, ballooning and akinesia of the basal and mid segments of the inferior wall.

Supplementary Video 3 Transthoracic echocardiography, apical three-chamber view. Hyperechogenicity, ballooning and akinesia of the basal and mid segments of the anteroseptal wall.

Supplementary Video 4 Initial cardiac MRI performed at day 3 after transfer to the nephrology intensive care unit. Left ventricular long axis view showed severe basal hypokinesia sparing the mid and apical cavity, which are in moderate hyperkinesia. This aspect is highly suggestive of a basal type of Takotsubo syndrome.

Supplementary Video 5 Cardiac MRI follow-up performed 1 month later showed complete resolution of wall motion abnormalities including complete recovery of the basal left ventricular wall motion.

Supplementary Figure 1 Timeline. I.V, intravenous; HELLP, hemolysis elevated liver enzymes and low platelets count; TTE, transthoracic echocardiography; ACE, angiotensin conversion enzyme; LVEF, left ventricular ejection fraction; TKS, Takotsubo syndrome; MRI, magnetic resonance imaging; cTnThs, cardiac high-sensitivity troponin I; cTnIhs, cardiac high-sensitivity troponin I; NT-proBNP, N-terminal prohormone of brain natriuretic peptide.
