# Supplementary figures and images for: Inverted Takotsubo Syndrome With HELLP Syndrome: A Case Report
Source: Front Cardiovasc Med. 2022 Mar 31;9:832098. doi: 10.3389/fcvm.2022.832098 (PMC9008274; doi:10.3389/fcvm.2022.832098)

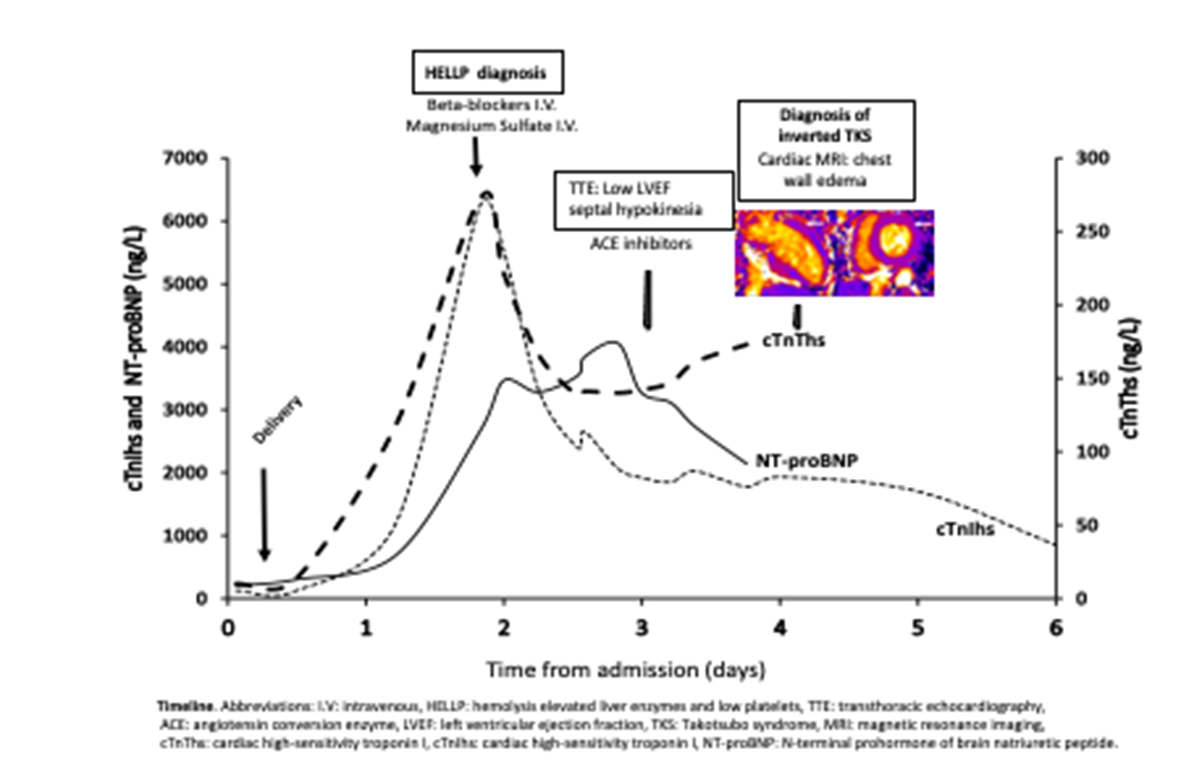

Supplement: Supplementary Figure 1 — Timeline. I.V, intravenous; HELLP, hemolysis elevated liver enzymes and low platelets count; TTE, transthoracic echocardiography; ACE, angiotensin conversion enzyme; LVEF, left ventricular ejection fraction; TKS, Takotsubo syndrome; MRI, magnetic resonance imaging; cTnThs, cardiac high-sensitivity troponin I; cTnIhs, cardiac high-sensitivity troponin I; NT-proBNP, N-terminal prohormone of brain natriuretic peptide. [file Image_1.tiff]
